# Validation of Airway Wall Measurements by Optical Coherence Tomography in Porcine Airways

**DOI:** 10.1371/journal.pone.0100145

**Published:** 2014-06-20

**Authors:** Anthony M. D. Lee, Miranda Kirby, Keishi Ohtani, Tara Candido, Rebecca Shalansky, Calum MacAulay, John English, Richard Finley, Stephen Lam, Harvey O. Coxson, Pierre Lane

**Affiliations:** 1 Department of Integrative Oncology - Imaging Unit, British Columbia Cancer Agency Research Centre, Vancouver, British Columbia, Canada; 2 Department of Radiology, Vancouver General Hospital, Vancouver, British Columbia, Canada; 3 Department of Pathology, Vancouver General Hospital, Vancouver, British Columbia, Canada; 4 Department of Surgery, Vancouver General Hospital, Vancouver, British Columbia, Canada; Mayo Clinic College of Medicine, United States of America

## Abstract

Examining and quantifying changes in airway morphology is critical for studying longitudinal pathogenesis and interventions in diseases such as chronic obstructive pulmonary disease and asthma. Here we present fiber-optic optical coherence tomography (OCT) as a nondestructive technique to precisely and accurately measure the 2-dimensional cross-sectional areas of airway wall substructure divided into the mucosa (WA_muc_), submucosa (WA_sub_), cartilage (WA_cart_), and the airway total wall area (WAt). Porcine lung airway specimens were dissected from freshly resected lung lobes (N = 10). Three-dimensional OCT imaging using a fiber-optic rotary-pullback probe was performed immediately on airways greater than 0.9 mm in diameter on the fresh airway specimens and subsequently on the same specimens post-formalin-fixation. The fixed specimens were serially sectioned and stained with H&E. OCT images carefully matched to selected sections stained with Movat’s pentachrome demonstrated that OCT effectively identifies airway epithelium, lamina propria, and cartilage. Selected H&E sections were digitally scanned and airway total wall areas were measured. Traced measurements of WA_muc_, WA_sub_, WA_cart_, and WA_t_ from OCT images of fresh specimens by two independent observers found there were no significant differences (p>0.05) between the observer’s measurements. The same wall area measurements from OCT images of formalin-fixed specimens found no significant differences for WA_sub_, WA_cart_ and WA_t_, and a small but significant difference for WA_muc_. Bland-Altman analysis indicated there were negligible biases between the observers for OCT wall area measurements in both fresh and formalin-fixed specimens. Bland-Altman analysis also indicated there was negligible bias between histology and OCT wall area measurements for both fresh and formalin-fixed specimens. We believe this study sets the groundwork for quantitatively monitoring pathogenesis and interventions in the airways using OCT.

## Introduction

Airway wall remodelling is a key component of chronic lung diseases such as chronic obstructive pulmonary disease (COPD) and asthma. Historically, COPD and asthma have been studied using surgical specimens and bronchial biopsies, with histopathology considered the ‘gold-standard’ for quantifying airway dimensions. While producing very valuable information, histology has a few key limitations. Histology requires the removal of tissue and is therefore limited in sampling, both in terms of the number of available samples for cross-sectional studies and in the application of the technique for longitudinal and interventional studies. Specimen shrinkage and distortion during histological processing steps also limit the accuracy of quantitative measurements [1]–[3]. Additionally, *ex vivo* specimens differ from the natural *in vivo* state of the lung because of the lack of surrounding supporting structures, blood flow, and ventilation. Therefore, to best understand both the pathogenesis of these diseases as well as to evaluate the effect of interventions, it is necessary to precisely and accurately quantify airways in their *in vivo* state.

Optical coherence tomography (OCT) is a relatively new imaging technique that can potentially overcome many of the aforementioned limitations for *in vivo* imaging of the airways. OCT is the optical analog of ultrasound and is ideally suited for imaging airways because the shorter wavelength of light compared to sound allows for higher resolution images to be obtained. Studies have shown that OCT can visualize cellular and extra-cellular structures at and below the tissue surface [4]–[6], and provide cross-sectional images of anatomic tissue structures at a spatial resolution approaching histology and a depth penetration of greater than1 mm. OCT imaging studies of *ex vivo* lung surgical samples has allowed precise co-registration with histopathology to identify airway wall components and precise pathological correlation [7]–[9].

Fiber optic OCT probes have also been developed for endoscopic use that have allowed *in vivo* imaging of the airways; studies have identified and correlated features in *in vivo* OCT imaging with neoplastic changes in the lung [10], [11]. In the interest of monitoring and quantifying COPD, *in vivo* studies have shown significant correlations between airway total wall area measured using OCT and computed tomography (CT) [12], but with its increased resolution, OCT may actually be more sensitive than CT. A related technique, anatomical OCT (aOCT) has also been used to accurately measure airway calibre [13] and to evaluate the elastic properties of the central airways in obstructive airway diseases [14]. In the former study, aOCT was used to quantitatively measure on a limited sample size the airway inner wall area and thickness. aOCT was also used to demonstrate dynamic imaging of airways under electric field stimulation of the airway smooth muscle.

With respect to evaluating airway remodelling in chronic lung diseases, there have been limited studies validating airway wall areas measured with OCT and comparing them with those from precisely matched histology. Here we present a study of ex vivo porcine airways and our objectives were to: 1) identify the sub-surface components of the airway wall relevant to airway disease, 2) measure OCT airway wall components and compare these measurements to the gold standard (histology), and, 3) evaluate the inter-observer reproducibility of OCT airway wall component measurements. Demonstrating these capabilities in an *ex vivo* setting should allow the extension of OCT imaging to accurately and precisely measure airway walls *in vivo*.

## Methods

### Porcine Lung Preparation

**Figure 1:**
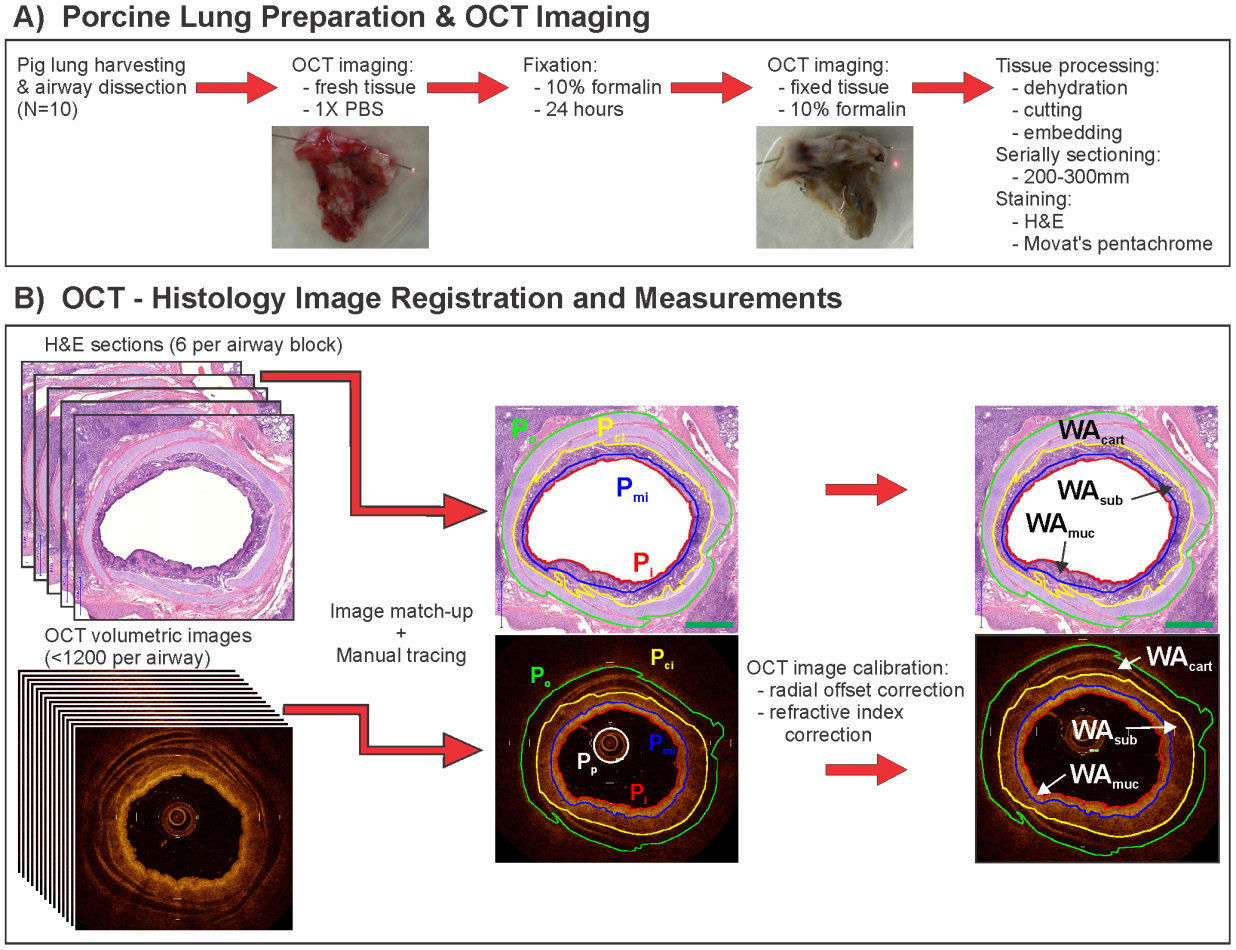
Correlating OCT imaging and histology. A) Experimental procedure for airway preparation and OCT imaging. B) Diagram outlining matching of H&E and OCT images, manual tracing of the morphological perimeters, and calculation of the airway wall area components. P_p_ = probe perimeter, P_i_ = luminal perimeter, P_mi_ = muscle inner perimeter, P_ci_ = cartilage inner perimeter, P_o_ = airway outer boundary perimeter. WA_muc_ = mucosal wall area, WA_sub_ = submucosal wall area, WA_cart_ = cartilage wall area. Scale bar = 1 mm.

Preparation of the *ex vivo* specimen and OCT imaging procedures are outlined in Figure 1A and described below. Fresh lungs were harvested from Yucatan miniature pigs (N = 10) immediately following euthanization. Two or three small to medium sized airways (>1 mm diameter, 3 to 5 cm long) were dissected from the lungs and marked with ink to provide orientation landmarks for imaging and histological preparation. The specimens were kept on ice for immediate use.

The airway specimens were rinsed with phosphate buffered saline (PBS) solution to remove mucous and blood. The entire fresh airway specimens were imaged while submerged under PBS to minimize probe-air and tissue-air interface reflections. Following overnight fixation in 10% formalin solution, the specimens were submerged in fresh 10% formalin solution and OCT imaged for a second time. The specimens were then dehydrated using standard histological procedures. Due to the dimensions of the embedding trays, airway specimens were cut into segments of 1 cm or shorter. Photographs and additional ink markers ensured correct ordering and orientation of the multiple airway segments prior to embedding into paraffin blocks. Selected blocks were serially sectioned with 200 um to 300 um spacing between adjacent sections and stained with haematoxylin and eosin (H&E). Two of the blocks were sectioned such that 2 slides were collected every 300 um. One slide from each of the 2-slide sets was stained with H&E. The second slide from the set was stained with Movat’s pentachrome to further differentiate tissue components. All slides were scanned at 20X using a Panoramic MIDI slide scanner (3DHistech Kft., Hungary).

### Ethics Statement

The donation of animal tissue was approved by the institutional review board at the University of British Columbia (A11-0374).

### OCT Imaging

A Lightlabs C7XR (St. Jude Medical, St. Paul, MN) swept-source OCT system was used for imaging. The specimens were imaged using 0.9 mm diameter rotary-pullback C7 Dragonfly catheters (St. Jude Medical, St. Paul, MN). Light exits the probe in the forward direction at an angle 68.5° relative to the probe axis in air. This system is centred around 1310 nm and provides axial resolution of approximately 15 µm and a section thickness of 5 µm. Three-dimensional volumetric images were acquired at 100 Hz frame rate and 2 to 5 mm/s pullback rate, providing an imaging pitch of 20–50 µm between frames.

### OCT - Histology Image Registration

The procedures for registering OCT and histology images and airway wall measurements is shown in Figure 1B. Six histology sections from a single airway block for each pig lung were selected for matching to the fresh and formalin-fixed OCT images. Sections were chosen over a range of airway calibre to give a representative sample of airways 0.9 mm to approximately 4.5 mm in diameter. The OCT images (up to 1200 per airway) were reviewed on a Lightlabs workstation and compared to the histology images viewed concurrently using Panoramic Viewer software (3DHistech Kft., Hungary). Anatomic landmarks such as airway branch points and cartilage were used as reference points along each airway to match OCT images and histology sections. Due to the greater rigidity of the formalin fixed airways, and thus closer resemblance to histology, the formalin-fixed OCT images were first matched to histology. The positioning of the formalin-fixed matched OCT images along the airways assisted in matching to the fresh OCT images.

### OCT Image Measurements

For each fresh and formalin-fixed OCT image in the matched sets, the following perimeters were traced using ImageJ software (NIH, Bestheda, MD) (abbreviations derived from [15]): the probe outer diameter (Pp), the luminal perimeter (Pi), the muscle inner perimeter (Pmi), the cartilage inner perimeter (Pci); and the airway outer boundary perimeter (Po). The airway outer boundary perimeter was taken to be outer surface of the furthest cartilage ring. Although visualized in OCT, we did not attempt to delineate the basement membrane perimeter (Pbm) as its displacement from Pi is not much greater than the resolution of the OCT images The OCT images were traced by two independent observers (Observers 1 & 2). In the rotary scanning geometry, OCT data is acquired in polar coordinates where *r* and *θ* correspond to the radial (A-line) and azimuthal directions respectively. Accurate transformation into the Cartesian representation first requires calibration of the *r*-axis relative to a known reference. Here we offset the *r*-axis for all histology matched OCT frames so that the area enclosed by Pp in the Cartesian OCT images is equal to the cross-sectional area of the 0.9 mm diameter probe. Radial calibration of the OCT images was made assuming a refractive index of 1.336 (the refractive index of water) for the PBS and formalin solutions between the probe and the luminal boundary, and a tissue refractive index of 1.38, the literature value reported for blender-homogenized porcine lung tissue at 632.8 nm [16], for everything beyond the luminal boundary. The refractive index-corrected tracings were used to calculate the areas Ai, Ami, Aci, and Ao corresponding to the traced perimeters Pi, Pmi, Pci, and Po respectively. Due to the slightly forward-looking probe geometry resulting in conical rather than planar cross-sectional OCT images, we multiplied the values of: Ai by a factor of sin(74.2°), the sine of the exit angle of light from the probe in PBS/formalin; and Ami, Aci, and Ao by a factor of the sin(74.7°), the sine of the exit angle of light from the probe in tissue. Three airway wall sub-compartment areas were calculated as differences with the mucosal wall area, WA_muc_ = Ami – Ai, containing the airway epithelium, basement membrane, and lamina propria; the submucosal wall area, WA_sub_ = A_ci_ – A_mi_, containing the airway smooth muscle and glands; and the cartilage wall area, WA_cart_ = A_o_ – A_ci_, containing the airway cartilage. The airway total wall area, WA_t_, was calculated as the sum of WA_muc_, WA_sub_, and WA_cart_.

### Histology Image Measurements

Analogously to the OCT perimeters, P_i_, P_mi_, P_ci_, and P_o_, were traced on the H&E stained histology images by a single observer (Observer 1). As artifacts during tissue processing creates considerable white space or voids in the histological slides especially adjacent to the airway cartilage, P_ci_ was traced to lie approximately halfway between the cartilage inner boundary and the submucosal outer boundary. The areas enclosed by the perimeters were used to generate the corresponding areas A_i_, A_mi_, A_ci_, and A_o_. Wall areas WA_muc_, WA_sub_, WA_cart_, and WA_t_ were calculated as for the OCT measurements. Additionally, image processing was used to mask out the white space from area (A_mi_, A_ci_, and A_o_) and all wall area measurements to yield the quantities A_mi_′, A_ci_′, A_o_′, WA_muc_′, WA_sub_′, WA_cart_′, and WA_t_′.

### Statistics

A paired two-tailed t-test with Holm-Bonferroni correction for multiple comparisons was performed for statistical comparison between the two observer’s OCT measurements using GraphPad Prism version 4.00 (GraphPad Software Inc, San Diego, CA, USA). The relationship between the two observers OCT measurements was determined using Pearson correlation coefficients (r); Bland-Altman analysis was also used to determine the agreement between observers OCT measurements using GraphPad Prism version 4.00. OCT measurement inter-observer reproducibility was calculated using the coefficient of variation (CV); CV was calculated as the pooled standard deviation (SD) of the two observers measurements divided by the pooled mean of the two observers measurements and multiplying the result by 100.

Airway wall areas (WA_muc_, WA_sub_, WA_cart_, and WA_t_) measured with 1) OCT on fresh specimens; 2) OCT on post-formalin fixed specimens; and 3) histology were compared pair-wise using a linear regression model with least squares optimization. The OCT measurements were pooled from both observers for the linear regression and Bland-Altman analysis.

## Results

A total of 60 H&E slide images and formalin-fixed OCT images from 10 different airway blocks were used for quantitative measurement of the airway total wall area.

### Identification and Manual Segmentation of Airway Wall Substructure

**Figure 2:**
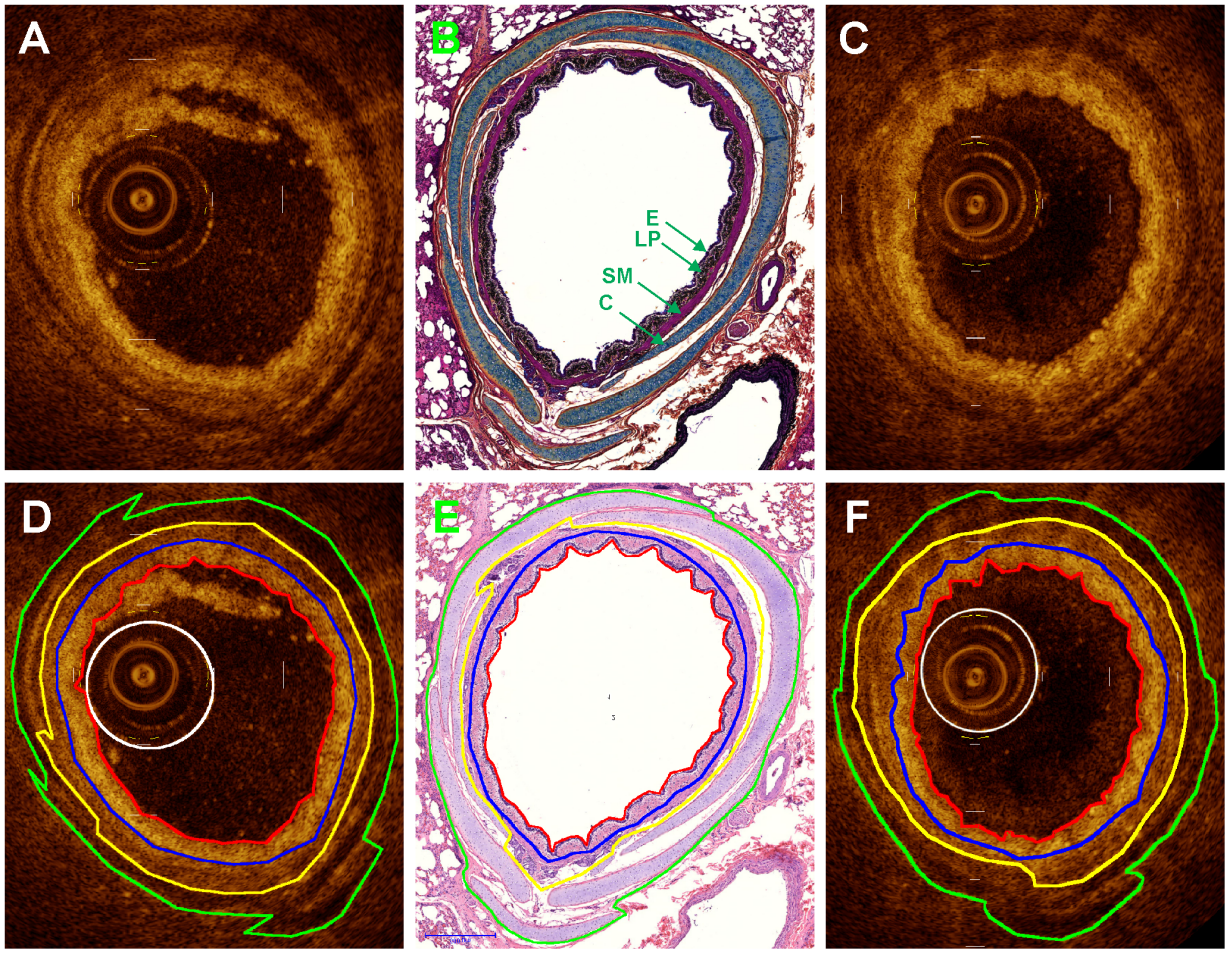
Matched and traced OCT and histology images of porcine airway. A, D) Fresh OCT images, B, E) H&E and Movat’s pentachrome photomicrographs respectively, C, F) Post-formalin fixed OCT images. Tracings: green = P_o_, yellow = P_ci_, blue = P_mi_, red = P_i_, white = P_p_. in B) E = epithelium, LP = lamina propria, SM = smooth muscle, C = cartilage.

An example matched set of fresh and post-formalin fixed OCT and H&E histology images are shown in Figure 2 with an adjacent Movat’s stained section also included for easier morphological identification. Movat’s pentachrome stain readily identifies sub-airway wall components such as the nuclei-dense epithelium and lamina propria (black), smooth muscle (purple) and cartilage (yellow-blue). The tracings of P_p_ (where applicable), P_i_, P_mi_, P_ci_, and P_o_ are also shown. Qualitatively, there are no gross differences in the general appearance and relative intensities of the airway wall sub-components between the fresh and formalin-fixed OCT images. The moderately intense scattering epithelium lies atop the very intensely scattering lamina propria. Just below the lamina propria lies a moderately intense band corresponding to the smooth muscle. The cartilage plates appear dark in OCT imaging. The extra body between the luminal perimeter and the probe perimeter in the fresh OCT image is coagulated blood that was not flushed from the airway prior to imaging.

### Inter-Observer Reproducibility of OCT Measurements

**Figure 3:**
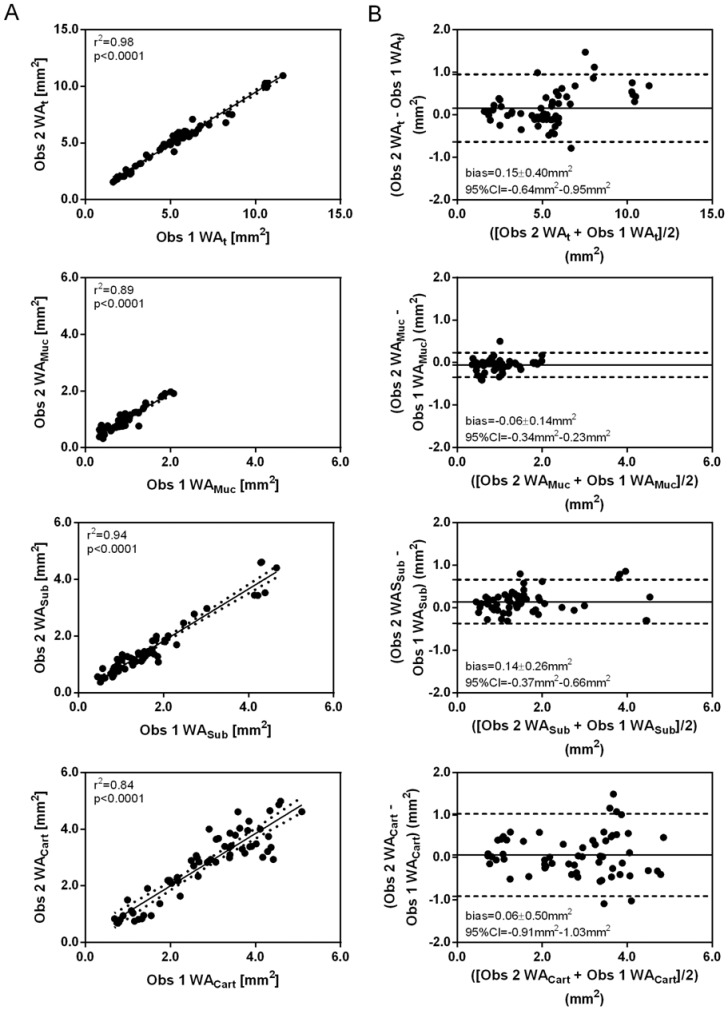
Correlation and Bland-Altman analysis for fresh OCT airway wall component measurements for two observers for all slices. A. There was a significant correlation between observer 1 and 2_t_ (r^2^ = 0.98, p<0.0001), WA_muc_ (r^2^ = 0.89, p<0.0001), WA_sub_ (r^2^ = 0.94, p<0.0001) and WA_cart_ (r^2^ = 0.84, p<0.0001). B. Bland-Altman analysis indicates a small bias between observers for WA_t_ (bias = −0.15±0.40, 95% CI = −0.64–0.95), WA_muc_ (bias = 0.06±0.14, 95% CI = −0.34–0.23), WA_sub_ (bias = 0.14±0.26, 95% CI = −0.37–0.66), WA_cart_ (bias = −0.06±0.50, 95% CI = −0.91–1.03). Solid lines represent the bias and dotted lines represent the 95% confidence intervals.

As shown in Table 1, there were no significant differences between the two observers for any of the OCT-fresh airway wall component or airway boundary measurements. Figure 3 shows the Pearson correlations and Bland-Altman plots between the two observers for OCT-fresh airway wall component measurements. For OCT-fixed measurements, there were strong and significant correlations between the two observers for WA_t_ (r^2^ = 0.98, p<0.0001), WA_muc_ (r^2^ = 0.89, p<0.0001), WA_sub_ (r^2^ = 0.94, p<0.0001) and WA_cart_ (r^2^ = 0.84, p<0.0001); Bland-Altman analysis indicated there was a negligible bias between the two observers for WA_t_ (bias = −0.15±0.40, 95% CI = −0.64–0.95), WA_muc_ (bias = 0.06±0.14, 95% CI = −0.34–0.23), WA_sub_ (bias = 0.14±0.26, 95% CI = −0.37–0.66), WA_cart_ (bias = −0.06±0.50, 95% CI = −0.91–1.03). The comparison between observers for post-formalin OCT measurements is provided in Text S1 in File S1.

**Table 1:** Comparison of Mean Whole Lung Airway Wall Component Measurements for Two Observers.

	Obs 1 (N = 10)	Obs 2 (N = 10)	Difference (±SD)	P-value
A_i_ mm^2^	3.12	3.09	−0.03 (0.12)	1.00
A_mi_ mm^2^	4.05	4.07	0.02 (0.07)	0.74
A_ci_ mm^2^	5.72	5.60	−0.12 (0.14)	0.14
A_o_ mm^2^	8.51	8.32	−0.19 (0.33)	0.66
WA_muc_ mm^2^	1.67	1.52	0.05 (0.11)	0.72
WA_sub_ mm^2^	2.79	2.73	−0.14 (0.15)	0.08
WA_cart_ mm^2^	5.39	5.24	−0.06 (0.39)	0.63
WA_t_ mm^2^	0.93	0.99	−0.15 (0.32)	0.85

Inter-observer reproducibility was moderately high for OCT-fresh measurements (WA_t_: CV = 5%, 95% CI = 3–8%; WA_muc_: CV = 9%, 95% CI = 6–16%; WA_sub_: CV = 9%, 95% CI = 6–16%; WA_cart_: CV = 10%, 95% CI = 7–17%). Inter-observer reproducibility for post-formalin OCT measurements is provided in Text S1 in File S1.

### Comparison of Measurements with OCT and Histology

**Figure 4:**
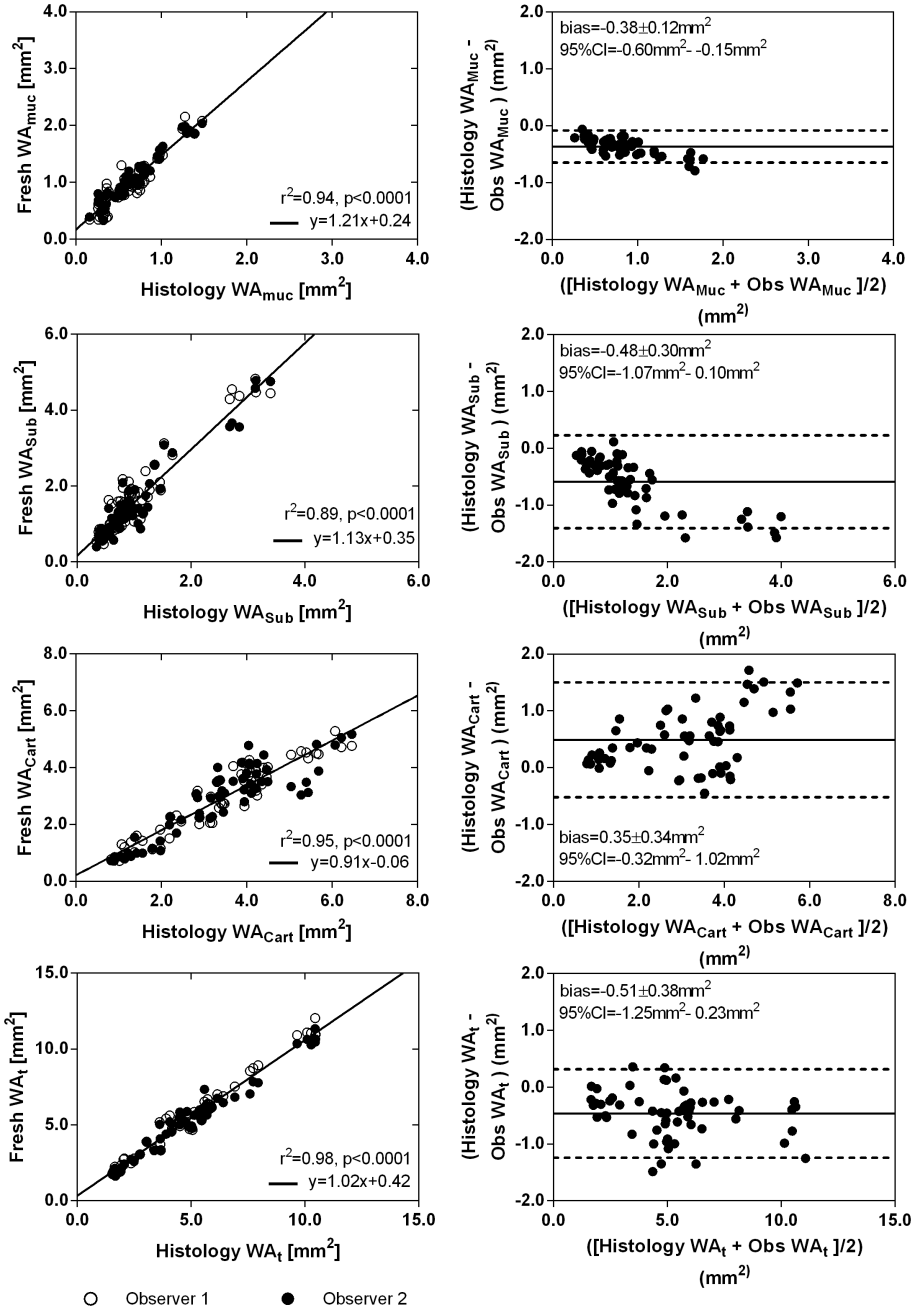
Fresh OCT airway wall component measurements versus histology. (A) Correlation plots for measurements of WA_muc_, WA_sub_, WA_cart_, and WA_t_ by both observers for fresh OCT imaging vs. histology (whitespace included). The solid lines are linear regression fits of the data and dotted lines are the regression fits of the data with the intercept of the model constrained to pass through the origin. (B) Bland-Altman analysis for WA_muc_, WA_sub_, WA_cart_, and WA_t_ measurements by both observers for fresh OCT imaging vs. histology. Solid lines represent the bias and dotted lines represent the 95% confidence intervals.

Figure 4 shows the linear regression and Bland-Altman analysis between measurements of WA_muc_, WA_sub_, WA_cart_, and WA_t_ obtained from fresh OCT imaging and those obtained from histology. WA_muc_, WA_sub_, and WA_t_ measurements were larger in fresh OCT imaging compared to histology as shown by the magnitude of the Bland-Altman bias (WA_muc_: bias = −0.36±0.14, 95% CI = −0.65− −0.08; WA_sub_: bias = −0.59±0.42, 95% CI = −1.41–0.23; WA_t_: bias = −0.46±0.40, 95% CI = −1.24–0.32), whereas WA_cart_ measures smaller than histology (bias = 0.49±0.52, 95% CI = −0.52–1.50). The linear regression results are shown in Figure 4, while Table 2 shows the slopes of the linear fits with standard error for measurements obtained from fresh OCT imaging versus histology; and post-formalin OCT imaging versus histology. Additionally, Table 2 shows the linear fit results for the comparison of post-formalin OCT imaging with fresh OCT imaging. Upon fixation, measurements of WA_muc_ remained approximately the same (slope = 0.90±0.03), WA_sub_ decrease (slope = 0.78±0.03), WA_cart_ increase (slope = 1.08±0.04), while the total wall area WA_t_ stays the same (slope = 0.98±0.02).

**Table 2:** Slopes of the linear fits with standard error for wall area component measurements between Fresh OCT, Post-Formalin OCT, and histology images for two observers.

	Fresh OCT vs. Histology	Post-Formalin OCT vs. Histology	Post-Formalin OCT vs. Fresh OCT
WA_muc_	1.30±0.05 (0.93)	1.21±0.04 (0.94)	0.90±0.03 (0.94)
WA_sub_	1.40±0.06 (0.92)	1.13±0.05 (0.89)	0.78±0.03 (0.92)
WA_cart_	0.79±0.04 (0.89)	0.91±0.03 (0.95)	1.08±0.04 (0.93)
WA_t_	1.03±0.02 (0.97)	1.02±0.02 (0.98)	0.98±0.02 (0.98)
WA_muc_′	1.31±0.05 (0.93)	1.21±0.04 (0.93)	
WA_sub_′	1.50±0.06 (0.92)	1.20±0.06 (0.88)	
WA_cart_′	0.82±0.04 (0.89)	0.95±0.03 (0.95)	
WA_t_’	1.08±0.02 (0.98)	1.06±0.02 (0.98)	

Wall areas denoted with a prime are for fits against histology wall areas with the whitespace area removed. R^2^ values are shown in parentheses.

## Discussion

The accurate analysis of airway dimensions is key to understanding chronic airway remodelling and its response to therapy. To date this analysis has been hampered by the destructive nature of histology on resected specimens and the limited resolution of CT scanning. In the current study we present data from a new non-destructive optical imaging technique that allows us to measure airway wall dimensions with micron scale resolution. Our data show that this technique produces images that allow investigators to visualize and measure the airway wall in a reproducible and reliable manner.

Our data show that OCT measurements of wall area between observers are significantly correlated and have negligible bias. There were also no significant differences between observers for the fresh OCT wall area (WA_x_) or area measurements (A_x_). Moderately high inter-observer reproducibility was found for OCT generated wall areas. Each wall area measurement is dependent on the accuracy of the two enclosing perimeters. For WA_muc_, the luminal perimeter (P_i_) is easily defined as the boundary between the imaging medium and the tissue, however, the delineation of the bottom of the highly scattering lamina propria (P_mi_) is more difficult and results in the relatively high value of CV = 9%. For WA_sub_, the inner cartilage perimeter (P_ci_) is well imaged as a sharp drop in scattering intensity, but the CV = 9% is primarily due to observer variability of P_mi_. The outer boundary of the cartilage (P_o_) is more difficult to discern in some cases because of the contrast falloff with distance from the probe, resulting in a relatively large CV = 10% for WA_cart_. When looking at WA_t_, the relatively low value of CV = 5% results because the P_o_ variability has a smaller effect on the total wall area. Interestingly, as seen in Text S1 in File S1, the CV values for post-formalin OCT imaging measurements of WA_muc_, WA_sub_, WA_cart_, and WA_t_ (CV = 7%, 6%, 5%, 2% respectively), are all less than for fresh OCT imaging, suggesting that formalin-fixation improves imaging contrast of the morphological perimeters. However, there was a small but significant difference in the mean values of the measured values of A_mi_ and WA_muc_ between observers.

By closely matching OCT image frames within the 3D data sets with histological sections, we compared airway wall measurements obtained from OCT and histology. Comparing post-formalin OCT imaging with fresh OCT imaging, we find that WA_muc_ is unaffected (slope = 0.99±0.01), however, WA_sub_ is smaller in post-formalin imaging by a factor of 0.89±0.02 while WA_cart_ is larger by a factor of 1.06±0.02. Overall for the total wall area, WA_t_, there is no difference (slope = 1.00±0.01) in the measured areas between fresh and post-formalin-fixation OCT imaging. The differential changes seen in WA_sub_ and WA_cart_ due to formalin fixation could be the result of several factors that may be a combination of: 1) the wall area subcomponents swelling or shrinking upon fixation; 2) the OCT imaging tissue contrast changing due to biochemical changes; 3) changing refractive indices of the tissue components.

The OCT wall area measurements, both fresh and post formalin, are significantly correlated with measurements from histology and the slopes of the regression lines reveal some interesting results regarding the swelling-shrinkage of the wall components. Fresh and post-formalin OCT imaging measures of WA_t_ are about the same, however, the airway wall subcomponents appear to be different with cartilage shrinking and the submucosa swelling. Removal of the whitespace from the histology images does change the amount of shrinkage and expansion observed for all wall areas, with the overall shrinkage of the total airway wall area WA_t_, reduced by a factor of 1.13±0.01 for histology compared to both fresh and post-formalin OCT imaging.

The differences between the OCT and histological measurements are due to either 1) artifacts due to histological processing or 2) OCT imaging artifacts. Histological processing artifacts (primarily the dehydration steps) may be the greater cause of the wall area measurement deviations from their true value for the following reasons. It is well documented that tissue shrinkage occurs during the preparation of histological slides; however, the amount of shrinkage can vary widely depending on the tissue type, and the processing procedure [1], [3]. For example, 35% area shrinkage has been reported for acrylic histological sections of porcine arteries [3]. Large shrinkage of the mucosal wall area WA_muc_, and submucosal wall area WA_sub_ is presumably primarily due to their high water content. Furthermore, sectioning can impart a distortion to the tissue that changes the aspect ratio of the tissue cross-sections [2], [17]. In addition, histological sectioning at a non-perpendicular angle to the long axis of the airway may be a potential source of difference between histology and OCT, and may also cause overestimation in histological area measurements. OCT imaging artifacts can be caused by non-uniform rotational distortion (NURD) in the spinning fiber-optic probe, improper tissue refractive index radial calibration, or refractive effects. However, in contrast to histology, these distortions are expected to be relatively small, and apart from NURD, can be corrected if accurate information about the refractive indices of the tissue specimen imaged are known. Apparent expansion of the cartilage wall area WA_cart_ in histology may be due in part to an incorrect value of the refractive index used for OCT reconstruction. However, corrections within the realm of reasonable refractive indices did not yield considerable differences to the expansion factors. Another possible explanation for the apparent WA_cart_ expansion in histology compared to OCT imaging is that the organization of cartilage plates can be modified whereby detachment from the submucosa causes the cartilage plates to relax to a larger, more open framework. Another potential OCT imaging artifact that may result in differences between histology and OCT airway measurements is mucous at the airway surface. Although we endeavored to remove mucous from the airway specimens in this study, for in vivo OCT imaging, any mucous at the airway surface during image acquisition may result in reduced depth penetration or refraction/distortion at curved surfaces. Although there are several factors that may lead to OCT image artifacts or lead to potential differences between histology and OCT, we must point out that the fact that the images were acquired using helical scanning is an unlikely source. Helical scanning is unlikely to cause differences between OCT and histology measurements as the imaging pitch of the helix (pitch = 20–50 µm) is comparable to the lateral resolution (25–30 µm).

For airway diseases such as asthma, it would be ideal if the airway smooth muscle (ASM) could be easily visualized and measured. The quantity measured here WA_sub_ contains both the ASM and glands. In these experiments, visualization of ASM by structural OCT imaging alone was variable. Further extensions of OCT such as endoscopic polarization-sensitive OCT [18] may have additional power to visualize ASM through its intrinsic birefringence.

The results of this *ex vivo* study show that OCT produces images that accurately identify airway wall substructure and that inter-observer measurements of airway wall subcomponents and total airway wall area are consistent. While OCT imaging was performed on airways submerged in PBS or formalin, it is reasonable to assume that the results we have shown here are translatable to *in vivo* OCT airway imaging. One reason for submerging the airways was to eliminate drying of the specimen which is not an issue in *in vivo* imaging. Imaging *in vivo* with air in the airway will likely yield similar imaging quality with the reduction in quality due to the higher reflection at the air-tissue interface offset by the reduced scattering of air compared to PBS and formalin. For *in vivo* OCT imaging in air, proper care will be needed to correct for the appropriate refractive indices and larger refractive imaging distortions. One of the major strengths of OCT is that, unlike histology, OCT is able to measure airways in their natural state that are ventilated, perfused with blood, and supported by surrounding structures. In an *in vivo* setting, blood perfusion may decrease depth penetration into the tissue further reducing the accuracy of the deeper airway wall component measurements. Nevertheless, these findings suggest OCT will be a very useful tool for the analysis of the airway wall to study *in vivo* both pathogenesis and response to intervention in airway diseases such as COPD and asthma.

However, it is important to note that for longitudinal and serial human studies it will be challenging to identify the same location within the airway tree. Importantly, to better understand how airway remodeling is modified by therapy or to determine how airway remodeling occurs over time during disease progression, the reproducibility of OCT for identifying and evaluating the same airway segment within the airway tree must be established. In this regard, we have previously performed a small pilot study in current or ex-smokers evaluating the OCT scan-rescan insertion reproducibility and demonstrated that insertion reproducibility is high in the middle lobe [25]. However, this is an important consideration for future studies and should be evaluated further in a larger number of subjects.

We must, however, acknowledge that this study is limited by several factors. One limitation of our study was including only two observers in our inter-observer reproducibility analysis. Including more observers would have strengthened our claims that OCT measurements have high inter-observer reproducibility. We also acknowledge that OCT is limited by depth penetrance. Depth penetration in OCT images is an important consideration because there is potential for the depth penetrance within the airway to be insufficient for identifying and measuring the airway wall in subjects with significant airway remodeling. However, it is important to note that a previous study evaluating OCT airway wall thickness in 44 current and ex-smokers with a range of airflow limitation did not report the inability to identify and measure the airway wall [12], and therefore this may not be an important consideration for evaluating airway remodeling in the more peripheral airways - the major site of airflow obstruction [19] in COPD. We must also acknowledge that this study is limited because OCT measurements were compared to histology measurements, which are known to have shrinkage artifacts, and therefore the true bias of these measurements cannot be determined. Acquiring the images from the fresh cut surface of the formalin fixed airways would have allowed for a direct comparison with the OCT measurements. However, the excellent correlation between OCT and histology suggests this technique does provide reliable measurements.

Other techniques have been used to measure airway wall changes in airway diseases. Multi-detector row CT scanners and new airway measurement algorithms have made it possible to obtain measurements of *in vivo* images of airways to approximately the 6^th^ generation [12], [20], [21]. However, the spatial resolution of CT is only on the order of 0.5 mm which means that it is still impossible to accurately measure the dimensions of small airways less than 2 mm in diameter, which are known to be the most important structures in chronic airflow restriction [22]. Furthermore, there is no standard CT imaging or analysis algorithm, making comparisons between studies difficult. Therefore, attention has turned to more indirect measurements of airways by examining the extent of hyper-inflated regions of the lung on expiration CT scans [23], so called gas trapping, or examining regions of the lung that are not ventilated using magnetic resonance imaging of hyperpolarized noble gases [24]. While these latter techniques have provided some useful information, they still do not directly measure the actual airway structure but only a consequence of airway remodelling. With its superior resolution, on the order of 10’s of microns, OCT is able to directly measure small airways less than 1 mm inn diameter and detect smaller changes. Furthermore, OCT does not involve the use of ionizing radiation which limits the number of CT scans that individual subjects can receive.

Summarizing, we have performed OCT imaging on 10 excised porcine airways and matched as close as possible these images with selected histological sections (N = 60) of the same specimens. Measurements by two independent observers showed that there were no significant differences in fresh OCT measurements of the mucosa, submucosa, cartilage, and total wall areas. Comparisons of wall area measurements between OCT and histology showed that OCT measures larger mucosal and submucosal wall areas and smaller cartilage wall areas. The airway total wall area with OCT on fresh and fixed airways were larger than the same measurements on histology. OCT is an important technique for the imaging of human airways and these findings indicate that it may provide valuable information on tissue changes caused by disease and response to intervention.

## Supporting Information

File S1 **Supporting information.** Text S1, Comparison of post-formalin OCT imaging and histology for two observers. Table S1, Comparison of Mean Whole Lung Airway Wall Component Measurements in Post-Formalin OCT Imaging for Two Observers. Figure S1, Correlation and Bland-Altman Analysis for post-formalin OCT Airway Wall Component Measurements for Two Observers for All Slices.
(DOCX)
