# Supplementary material for: Validation of Airway Wall Measurements by Optical Coherence Tomography in Porcine Airways
Source: PLoS One. 2014 Jun 20;9(6):e100145. doi: 10.1371/journal.pone.0100145 (PMC4064993; doi:10.1371/journal.pone.0100145)
Supplement: File S1 — Supporting information. Text S1, Comparison of post-formalin OCT imaging and histology for two observers. Table S1, Comparison of Mean Whole Lung Airway Wall Component Measurements in Post-Formalin OCT Imaging for Two Observers. Figure S1, Correlation and Bland-Altman Analysis for post-formalin OCT Airway Wall Component Measurements for Two Observers for All Slices. (DOCX) [file pone.0100145.s001.docx]

**Text S1.** Comparison of post-formalin OCT imaging and histology for two observers.

As shown in Table S1, there was a significant difference between the observers for A_mi_ (p=0.02) and WA_muc­_ (p=0.02), but not for any other airway wall component or boundary layers. Figure S1 shows the Pearson correlations and Bland-Altman plots between the two observers for OCT-fixed airway wall component measurements. For OCT-fixed measurements, there were strong and significant correlations between the two observers for WA_t_ (r^2^=0.98, p<0.0001), WA_muc_ (r^2^=0.94, p<0.0001), WA_sub_ (r^2^=0.95, p<0.0001) and WA_cart_ (r^2^=0.93, p<0.0001); Bland-Altman analysis indicated there was a negligible bias between the two observers for WA_t_ (bias = -0.08±0.30, 95% CI=-0.49-0.66), WA_muc_ (bias = 0.08±0.10, 95% CI=-0.27-0.11), WA_sub_ (bias = 0.09±0.20, 95% CI=-0.30-0.48), WA_cart_ (bias = -0.07±0.37, 95% CI=-0.65-0.79).

Inter-observer reproducibility was high for OCT-fixed measurements (WA_t_: CV=2%, 95% CI=1-3%; WA_muc_: CV=7%, 95% CI=5-13%; WA_sub_: CV=6%, 95% CI=4-11%; WA_cart_: CV=5%, 95% CI=3-8%).

**Table S1.** Comparison of Mean Whole Lung Airway Wall Component Measurements in Post-Formalin OCT Imaging for Two Observers

|  | **Obs 1 (N=10)** | **Obs 2 (N=10)** | **Difference (±SD)** | **P-value** |
| --- | --- | --- | --- | --- |
| A_i_ mm^2^ | 2.83 | 2.82 | -0.002 (0.05) | 0.90 |
| A_mi_ mm^2^ | 3.76 | 3.83 | 0.07 (0.05) | 0.02 |
| A_ci_ mm^2^ | 5.30 | 5.28 | -0.02 (0.13) | 1.00 |
| A_o_ mm^2^ | 8.23 | 8.14 | -0.09 (0.12) | 0.16 |
| WA_t_ mm^2^ | 5.40 | 5.32 | -0.09 (0.10) | 0.15 |
| WA_muc_ mm^2^ | 0.93 | 1.01 | 0.08 (0.06) | 0.02 |
| WA_sub_ mm^2^ | 1.54 | 1.45 | -0.09 (0.11) | 0.12 |
| WA_cart_ mm^2^ | 2.93 | 2.86 | -0.07 (0.19) | 1.00 |

**
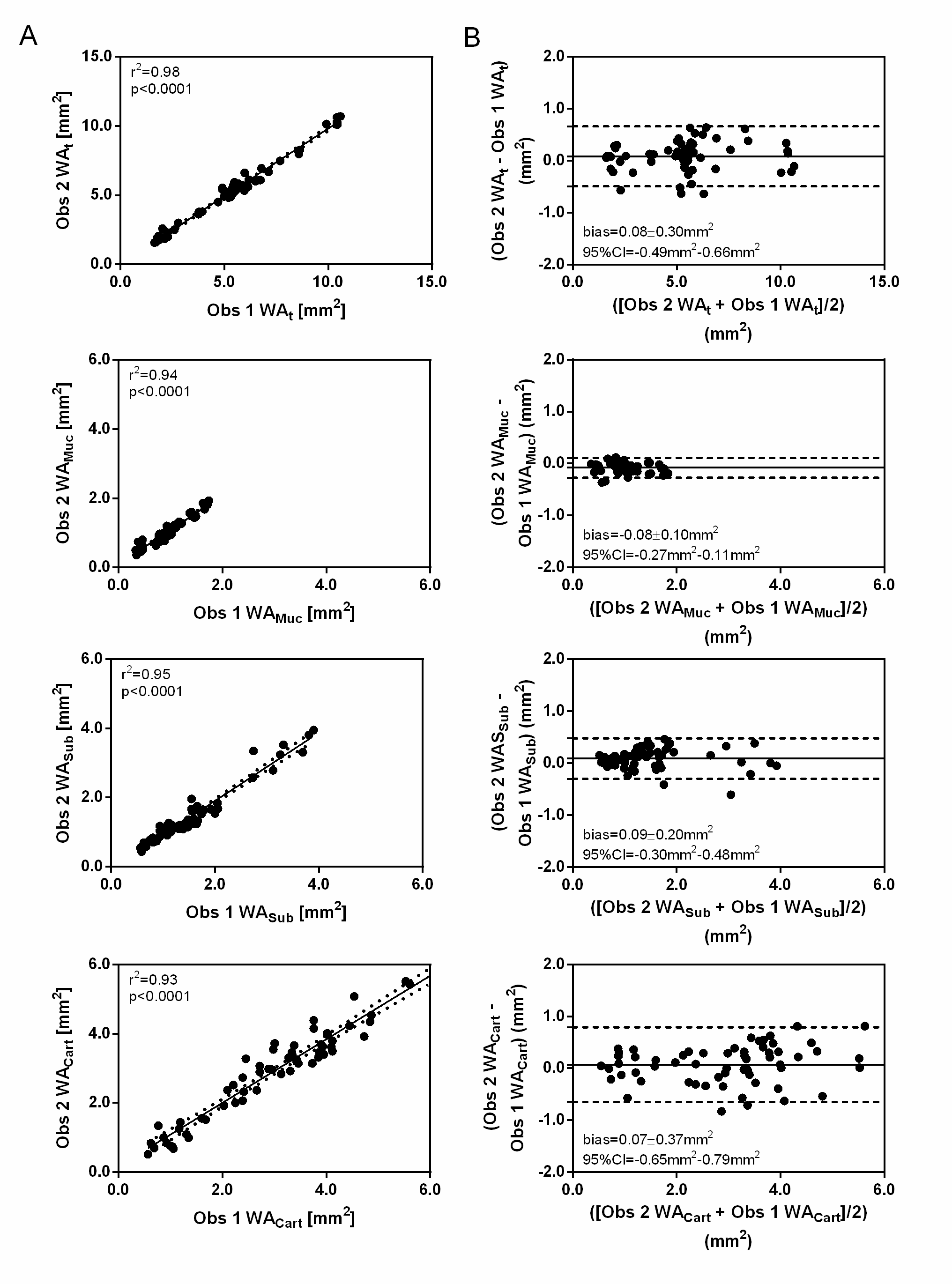
Figure S1.** Correlation and Bland-Altman Analysis for post-formalin OCT Airway Wall Component Measurements for Two Observers for All Slices

1. There was a significant correlation between observer 1 and 2 for OCT WA_t_ (r^2^=0.98, p<0.0001), WA_muc_ (r^2^=0.94, p<0.0001), WA_sub_ (r^2^=0.95, p<0.0001) and WA_cart_ (r^2^=0.93, p<0.0001).
2. Bland-Altman analysis indicates a negligible bias between observers for WA_t_ (bias = -0.08±0.30, 95% CI=-0.49-0.66), WA_muc_ (bias = 0.08±0.10, 95% CI=-0.27-0.11), WA_sub_ (bias = 0.09±0.20, 95% CI=-0.30-0.48), WA_cart_ (bias = -0.07±0.37, 95% CI=-0.65-0.79).

Solid lines represent the bias and dotted lines represent the 95% confidence intervals
